# Analysis of Active Compounds Using Target Protein Cofilin―Cucurbitacins in Cytotoxic Plant *Bryonia cretica*

**DOI:** 10.3390/toxins14030212

**Published:** 2022-03-16

**Authors:** Souichi Nakashima, Yoshimi Oda, Moeko Morita, Ayako Ohta, Toshio Morikawa, Hisashi Matsuda, Seikou Nakamura

**Affiliations:** 1 Department of Pharmacognosy, Kyoto Pharmaceutical University, Kyoto 607-8412, Japan; oda@nthhenna.co.jp (Y.O.); allpaqachan@gmail.com (M.M.); a-113.nmt@ezweb.ne.jp (A.O.); matsuda_134@outlook.jp (H.M.); naka@mb.kyoto-phu.ac.jp (S.N.); 2 NPR Medical Resource Laboratory, Kyoto 604-0924, Japan; 3 Pharmaceutical Research and Technology Institute, Kindai University, Osaka 577-8502, Japan; morikawa@kindai.ac.jp

**Keywords:** isolation with target protein, cofilin, cucurbitacin, isocucurbitacin D

## Abstract

We examined a two-step target protein binding strategy that uses cofilin as the target protein to analyze the active constituents in *Bryonia cretica*. In the first step, we prepared the target protein, and used it to analyze the compounds binding to it in the second step. We used the methanolic extract of *B. cretica* as a library of possible active compounds. We conducted LC–MS analysis using information from our previous study. The peaks in the HPLC profile were identified as cucurbitacin D, isocucurbitacin D, and cucurbitacin I. As far as we know, there is no known study of the activity of isocucurbitacin D in this research field. Therefore, we examined the effects of isocucurbitacin D on cell proliferation and cofilin protein in human fibrosarcoma cell line HT1080 to confirm the effectiveness of this strategy. The cytotoxicity assay, the fibrous/globular actin ratio assay, and the immunoblotting analysis revealed that isocucurbitacin D showed a cytotoxic effect with disruption of target protein cofilin. The target protein binding strategy is a direct and straightforward method for finding new drug seeds from crude sources, such as natural plant extracts.

## 1. Introduction

Cucurbitacins, which are a kind of triterpene from natural products, are known to exhibit strong cytotoxic and/or anti-proliferative effects on several cell lines [1]. We previously reported that cucurbitacin E showed anti-proliferative effects by binding target protein cofilin, which regulates the depolymerization of actin, a cell cytoskeleton [2]. Some researchers have reported and reviewed the effects of cucurbitacin E and its derivatives on cofilin [3,4,5], and their findings are in agreement with our study. In this regard, cofilin may be an important target of these types of compounds.

Cofilin plays an important role in the survival of several cancer cell lines. The ROCK/LIMK/cofilin signaling pathway in cancer cells is one of the important targets of cancer treatment. Many studies on drugs that show effects on cofilin and/or LIMK inhibitors have been reported [6]. However, studies on the relationships between the effects of such drugs and these proteins are lacking. We previously reported the isolation of several cucurbitacins from *Bryonia cretica*, Linné [1]. The isolation yield of cucurbitacin E (0.0085%) suggested the existence of other cucurbitacins with strong cytotoxicity. Therefore, to acquire more detailed information on the effects of cucurbitacins on cofilin, in this study, we tried to analyze the constituents of *B. cretica* affect to cofilin, by using novel methods that are based on the principle of target protein and drug combination. We also investigated the effects of the analyzed compounds, especially on the role of cofilin.

## 2. Results and Discussions

### 2.1. Preparation of Anti-Cofilin Antibody Binding Beads

**Figure 1:**
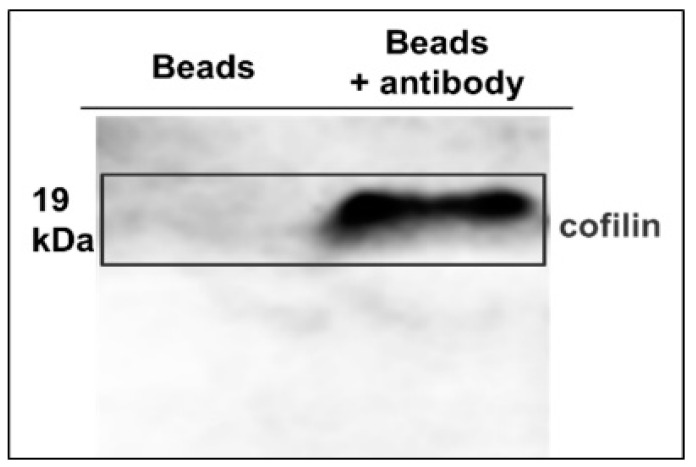
Confirmation of existence of cofilin by immunoblotting analysis. Left column: extracted with beads only; right column: extracted with anti-cofilin antibody binding beads.

First, we prepared cofilin from cultured HT1080 cells. To obtain the protein, we prepared anti-cofilin antibody binding beads using SH-labeled polystyrene microspheres, which are employed originally in flow cytometry assay. Anti-cofilin antibodies were attached to *N*-[(4-maleimidomethyl)cyclohexylcarbonyloxy]sulfosuccinimide (sulfo-SMCC). Next, the anti-cofilin antibodies attached to sulfo-SMCC were bound to SH-labeled beads after reduction by dithiothreitol (DTT). The binding of anti-cofilin antibodies to the SH-labeled beads was confirmed by flow cytometry. After mixing with cell lysate for 1 h, cofilin bound to the beads was collected by centrifugation. Then, cofilin was cleaved off with a glycine—HCl buffer (0.1 M, pH 3.0). Whether the collected proteins contained cofilin or not was confirmed by immunoblotting analysis (Figure 1).

Next, we prepared cofilin binding beads using the strategy described above. After preparing cofilin binding beads, we mixed the *B. cretica* methanolic extract and the beads for 1 h. After washing with a binding buffer, we separated the bound components by treatment with ethanol at 100 °C. *B. cretica* methanolic extract and its constituents were prepared and reported in our previous study [1].

### 2.2. LC-MS Analysis

**Figure 2:**
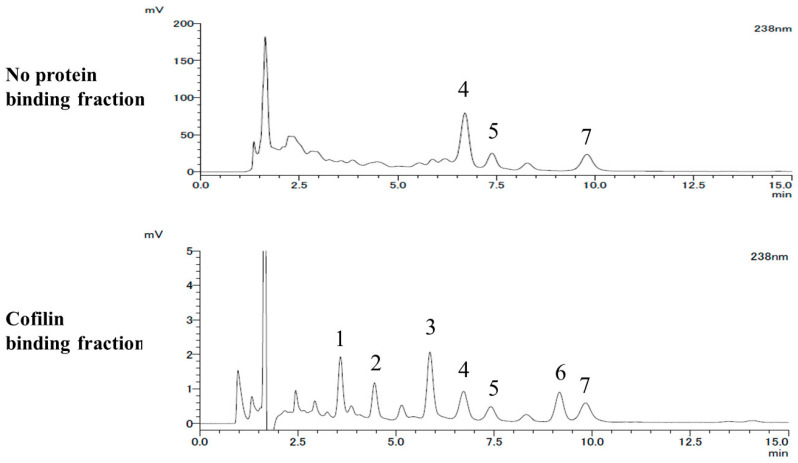
HPLC profiles of cofilin binding fraction and no protein binding fraction.

**Figure 3:**
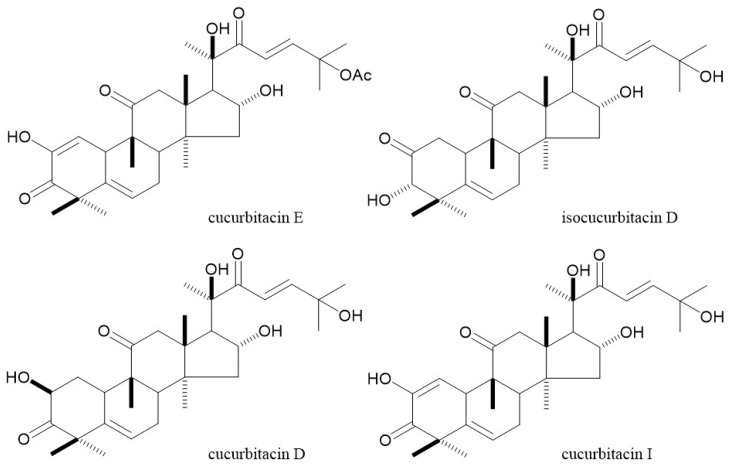
Chemical structures of four cucurbitacins.

In order to estimate and find the compounds binding to cofilin, we analyzed the HPLC profiles of the cofilin binding fraction and the no protein binding fraction from the *B. cretica* methanolic extract and found seven peaks in the cofilin binding fraction. (Table 1 and Figure 2) Among them, peak no. 4 was assigned to cucurbitacin D and peak no. 5 was assigned to isocucurbitacin D on the basis of LC-MS analysis with reference to previous reports [7,8] and appropriate modifications. The LC-MS spectra of the compounds corresponding to nos. 4 and 5 were indicated at *m*/*z* 561 [M+HCOOH-H]^−^. The compound corresponding to HPLC peak no. 7 was also estimated as cucurbitacin I, referring to the MS spectra, *m*/*z* 559 [M+HCOOH−H]^-^. The compound corresponding to the HPLC peak nos. 4, 5, and 7 were identified as cucurbitacin D, isocucurbitacin D and cucurbitacin I by the ^1^H and ^13^C NMR spectra, comparing with reference data (Figure 3) [9,10].

In addition, the compound corresponding to HPLC peak no. 2 was observed at *m*/*z* 579 and the other one corresponding to no. 3 was detected at *m*/*z* 166, 291, and 293, in negative ion mode analysis. The compound corresponding to HPLC peak no. 6 was also observed at *m*/*z* 221 in positive ion mode analysis. Unfortunately, the compounds corresponding to peak nos. 1–3 and 6 could not be identified because of their high degradability. Comparison of the HPLC profiles indicated that peak no. 5 in the cofilin binding fraction HPLC profile was approximately 1.63 (= 50.8/31.2) times larger (*p* < 0.01) than that in the no protein binding fraction HPLC profile, relative to peak no. 4. In addition, peak no. 7 corresponding to cucurbitacin I was 1.97 (= 95.7/48.5) times larger (*p* < 0.01) than that in the no protein binding fraction HPLC profile. From the results, we defined isocucurbitacin D and cucurbitacin I as the possible selective cofilin binding constituents in *B. cretica* methanolic extract. There are many reports of the cytotoxicity of cucurbitacin I and its modes of action. However, there is no detailed study on the activity of isocucurbitacin D in this research field.

### 2.3. Anti-Proliferative Effects

To confirm the effect of isocucurbitacin D on cofilin, we tested the anti-proliferative effect of isocucurbitacin D on HT1080 cells. As depicted in Table 2, isocucurbitacin D showed a significant anti-proliferative effect on the cells. The IC_50_ values did not change significantly from 24 to 72 h (0.64–0.71 μM). Cucurbitacin E as a positive control showed an anti-proliferative effect (IC_50_ after 72 h treatment: 40 nM).

### 2.4. Fibrous to Globular Actin Ratio in HT1080

Next, we examined the effect of isocucurbitacin D on the polymerization rate of actin in HT1080 cells. To clarify the polymerization rate, fibrous (filament) to globular (monomer) actin ratio (F/G ratio) was measured. As shown in Table 3, the F/G ratio decreased as isocucurbitacin D concentration increased. Because activated cofilin binds and depolymerizes actin filament, this triterpene has the possibility to activate or inhibit the inactivation of cofilin protein.

### 2.5. Effects of Isocucurbitacin D on the Phosphorylation of Cofilin

Cofilin is activated by de-phosphorylation (Ser3) and inactivated by phosphorylation (Ser3). Because it was reported that cucurbitacin E reduced phosphorylated- (p-) cofilin levels in a previous study, we examined the effect of isocucurbitacin D on the levels of p-cofilin and its kinase, protein p-LIMK1/2. As shown in Table 4 and Figure S1, isocucurbitacin D reduced p-cofilin levels and increased p-LIMK1/2 levels. These findings mean that isocucurbitacin D binds cofilin and inhibits cofilin phosphorylation, or accelerates the de-phosphorylation of p-cofilin. In other words, isocucurbitacin D possibly inhibits the phosphorylation of substrate in the enzyme reaction by binding to the substrate. This is one of the interesting points in this study. In addition, isocucurbitacin D is expected to accelerate the de-polymerization of actin, leading to anti-proliferative and/or cytotoxic effect, with this data.

## 3. Conclusions

In conclusion, using the target protein cofilin binding strategy, we found that isocucurbitacin D isolated from the methanolic extract of *Bryonia cretica* is a new inhibitor of cofilin. The peaks in the LC-MS profiles were identified as cucurbitacin D, isocucurbitacin D, and cucurbitacin I with ^1^H and ^13^C NMR spectra. Because there is no detailed study on the activity of isocucurbitacin D, we examined the effects of isocucurbitacin D on the proliferation of HT1080 cells and the expression of cofilin. The cytotoxicity assay, the F/G ratio assay, and immunoblotting analysis revealed that isocucurbitacin D showed cytotoxic effects with the disruption of target protein cofilin.

Several studies on compounds from natural resources such as plant extracts have been conducted with bioassay-guided physical separation and isolation. In this study, our objective was to find drug seeds using a target protein. Although further studies on the unknown peaks in the HPLC profile are required, this straightforward method shows potential for the direct determination of active constituents. We hope our findings contribute to the development of a new research tool for cell migration, cell proliferation, and cell motility studies.

## 4. Materials and Methods

### 4.1. Preparation of Anti-Cofilin Antibody Binding Beads

BD™ Cytometric Bead Array (CBA) Functional Bead A9 (BD Biosciences, San Jose, CA, USA) was used as the carrier in this study. The free NH_2_ group of anti-cofilin antibody (Cell Signaling Technology Japan, K.K., Tokyo, Japan) was reacted with sulfo-SMCC in 0.1 M Tris buffer (pH 7.5). After activation with 25 mM DTT, the exposed sulfhydryl groups of the beads were conjugated with the maleimide end of sulfo-SMCC linker in 0.1 M phosphate buffer (binding buffer; pH 6.0). Cofilin binding beads and *B. cretica* methanolic extract were mixed for 1 h and washed with binding buffer using centrifugation at 10,000× *g* for 10 min. The bound components were dissociated with ethanol at 100 °C. Other chemicals were purchased from FUJIFILM Wako Pure Chemical Co., Ltd. (Osaka, Japan).

### 4.2. LC-MS Analysis

All of the compounds were analyzed using a Nexera UHPLC system (Shimadzu Corporation, Kyoto, Japan) and an LCMS-8040 tandem quadrupole mass spectrometer. The instruments were operated with LabSolutions LCMS Ver.5.6. The Nexera UHPLC system was equipped with a system controller (CBM-20A), two pumps (LC-20A), an autosampler (SIL-20AC), a column heater (CTO-20AC), and a degasser (DGU-20A3R). Chromatographic separation was performed on a Cosmosil MS-II column (5 μm particle size, 2.0 mm i.d. × 150 mm, Nacalai Tesque, Inc., Kyoto, Japan) and other conditions were as follows: column temperature: 40 °C; mobile phase: 35% acetonitrile and 65% H_2_O; pH: 3.0 (adjusted with formic acid); flow rate: 0.2 mL/min; injection volume: 2.0 μL; detection wavelength: 238 nm. MS conditions were optimized, as follows: nebulizer gas flow, 3 L/min; drying gas flow, 15 L/min; desolvation line temperature, 250 °C; heat block temperature, 400 °C. ^1^H and ^13^C NMR spectra were detected by JEOL JNM-LA500 (500 MHz) (JEOL Ltd., Tokyo, Japan).

### 4.3. Cell Culture

Human fibrosarcoma cell line HT1080 (Cell No. JCRB9113) was obtained from the Japanese Collection of Research Bioresources Cell Bank (Osaka, Japan). The cells were maintained in Minimum Essential Medium Eagle [MEM; Sigma-Aldrich (St. Louis, MO, USA)] supplemented with 10% FBS [Thermo Fisher Scientific (Waltham, MA, USA)], 100 U/mL penicillin, and 100 μg/mL streptomycin at 37 °C in 5% CO_2_/air. The cells were harvested after incubation in phosphate-buffered saline (PBS) containing 1 mM EDTA and 0.25% trypsin for ca. 5 min at 37 °C and were used for the subsequent bioassays. A 96-well microplate was purchased from AGC Techno Glass Co., Ltd. (Shizuoka, Japan).

### 4.4. Assay for Anti-Proliferative Effects

Cell viability was assessed according to our previous reports [1,2] with slight modifications. The cells (5.0 × 10^3^ cells/100 μL/well) were seeded in a 96-well microplate and pre-cultured for 24 h (5% CO_2_, 37 °C). Then, the test samples were added. After incubation for 23, 47, and 71 h (5% CO_2_, 37 °C), 10 μL of MTT (0.5% *w/v*) was added. After incubation for 1 h, the produced MTT formazan was dissolved in 100 µL of 2-propanol containing 0.04 M HCl and the absorbance was measured with a microplate reader (SH-1000, CORONA ELECTRIC; measurement wavelength: 570 nm, reference wavelength: 655 nm). (Inhibition (%) = 100 − 100 × A/B, where A and B are the optical densities of the test-sample- and vehicle-treated groups, respectively.) The test sample was dissolved in DMSO and added to the medium (final concentration of DMSO: 0.1%). One-way analysis of variance followed by Dunnett’s test was used for statistical analysis.

### 4.5. Measurement of Fibrous to Globular Actin Ratio in HT1080

HT1080 cells (1.0 × 10^4^ cells/100 μL/well) were seeded in a 96-well microplate and incubated with test compounds for 24 h at 37 °C. The cells were washed three times with PBS and fixed with 4% formaldehyde in PBS for 15 min. Then, the cells were permeabilized with ice-cold methanol and blocked with Blocking One (Nacalai Tesque, Inc., Kyoto, Japan) for 30 min. After washing again with PBS, the cells were incubated for 15 min at r.t. in the dark with AlexaFluor^®^ 350 phalloidin (Thermo Fisher Scientific) in PBS. The cells were then incubated for 15 min at r.t. in the dark with deoxyribonuclease Alexa Fluor^®^ 488 conjugate (Thermo Fisher Scientific) in PBS. After the second instance of washing with PBS, the fluorescence intensity of each well was measured with a microplate reader (ex: 355 nm, em: 405 nm and ex: 480 nm, em: 520 nm, FLUOstar OPTIMA, BMG LABTECH). One-way analysis of variance followed by Dunnett’s test was used for statistical analysis.

### 4.6. Immunoblotting

Immunoblotting assay was conducted according to our previous report [11] with slight modifications. The primary antibodies, anti-cofilin (rabbit IgG), anti-p-LIMK1/2 (rabbit IgG), and anti-β-actin (rabbit IgG), were diluted 1000 times before use. The secondary antibody, anti-rabbit IgG antibody (donkey, peroxidase conjugated), was diluted 5000 times before use. All the antibodies were purchased from Cell Signaling Technology Japan K.K., Tokyo, Japan. LAS-4000 mini (FUJIFILM Corp., Tokyo, Japan) was used to measure and quantify the protein levels. The Kruskal–Wallis test, followed by Fisher’s exact test, was used for statistical analysis.

## Figures and Tables

**Table 1:** Peak area ratios of cofilin binding constituents relative to cucurbitacin D.

	Peak Area Ratio Relative to Cucurbitacin D		
	cuD/cuD	isocuD/cuD	cuI/cuD
No protein binding fraction	100.0 ± 0.2	31.2 ± 0.1	48.5 ± 0.2
Cofilin binding fraction	100.0 ± 1.4	50.8 ± 0.3	95.7 ± 0.9

Values are shown as means ± S.E.M. (*n* = 4). cuD: cucurbitacin D, isocuD: isocucurbitacin D, cuI: cucurbitacin I.

**Table 2:** Anti-proliferative effects of isocucurbitacin D on HT1080 cells.

	Conc. (μM)	Inhibition (%)						IC_50_ (μM)
		0	0.2	0.4	0.6	0.8	1.0	
24 h		100.0 ± 4.7	89.8 ± 1.8	75.8 ± 2.5 **	63.6 ± 2.2 **	30.2 ± 2.2 **	29.0 ± 1.0 **	0.66
48 h		100.0 ± 0.8	94.2 ± 1.5	75.8 ± 2.9 **	63.4 ± 1.8 **	26.3 ± 1.2 **	20.8 ± 0.6 **	0.64
72 h		100.0 ± 2.4	106.3 ± 4.4	98.2 ± 4.9	87.9 ± 7.8	25.1 ± 1.7 **	15.4 ± 0.1 **	0.71

Values are shown as means ± S.E.M. (*n* = 4). Significantly different from the control group, ** *p* < 0.01.

**Table 3:** Effects of isocucurbitacin D on fibrous (F) to globular (G) actin ratio in HT1080 cells.

Conc. (μM)	F/G Ratio (%)				
	0	0.01	0.1	1	10
	100.0 ± 9.3	74.1 ± 10.8	65.8 ± 6.5 **	77.1 ± 7.7	60.0 ± 16.1

Values are shown as means ± S.E.M. (*n* = 4). Significantly different from the control group, ** *p* < 0.01.

**Table 4:** Effects of isocucurbitacin D on p-cofilin/β-actin, p-LIMK1/β-actin, and p-LIMK2/β-actin levels.

Conc. (nM)	Expression Ratio (%)				
	0	1	10	100	1000
p-cofilin/β-actin	100	81.2 *	82.2 *	73.3 **	61.7 **
p-LIMK1/β-actin	100	161.6 **	211.4 **	161.5 **	131.1 *
p-LIMK2/β-actin	100	140.4 *	164.2 **	149.1 **	85.6

Values are shown as means (*n* = 2). Significantly different from the control group, * *p* < 0.05, ** *p* < 0.01.

## Data Availability

Not applicable.

## Supplementary Materials

The following supporting information can be downloaded at: https://www.mdpi.com/article/10.3390/toxins14030212/s1, Figure S1: Effects of isocucurbitacin D on p-cofilin/β-actin, p-LIMK1/β-actin, and p-LIMK2/β-actin levels.
