# Supplementary material for: Analysis of Active Compounds Using Target Protein Cofilin―Cucurbitacins in Cytotoxic Plant Bryonia cretica
Source: Toxins (Basel). 2022 Mar 16;14(3):212. doi: 10.3390/toxins14030212 (PMC8955846; doi:10.3390/toxins14030212)
Supplement: Supplementary file 1 [file toxins-14-00212-s001.zip › toxins-1620484-Supplementary.pdf]

toxins

MDPI

Communication

Analysis of Active Compounds Using Target Protein Cofilin—Cucurbitacins in Cytotoxic Plant *Bryonia cretica*

Souichi Nakashima, Yoshimi Oda, Moeko Morita, Ayako Ohta, Toshio Morikawa, Hisashi Matsuda and Seikou Nakamura

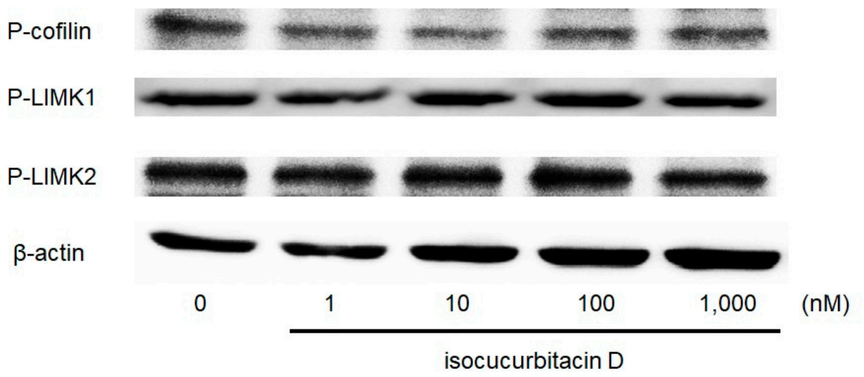

Figure S1. Effects of isocucurbitacin D on p-cofilin/β-actin, p-LIMK1/β-actin, and p-LIMK2/β-actin levels.
